# Dynamic corrosion behavior of superhydrophobic surfaces

**DOI:** 10.1039/c8ra05200j

**Published:** 2018-08-20

**Authors:** C. Q. Li, M. Y. Zhu, J. F. Ou, Y. L. Lu, F. J. Wang, W. Li

**Affiliations:** School of Materials and Engineering, Jiangsu University of Technology Changzhou 213001 P. R. China oujunfei_1982@163.com

## Abstract

For superhydrophobic surfaces immersed in water, a thin layer of air could be entrapped in the solid/liquid interface. This air may hinder the diffusion of dissolved corrosive species (such as Cl^−^ ions in water) to the metallic substrate and, consequently, protect the metal from corrosion. However, in the dynamic water, the relative motion between the solid and the liquid would labilize the entrapped air and, consequently, decrease the corrosion resistance. In this work, to clarify the role of water flow velocity in such corrosion behavior, a superhydrophobic surface on aluminum substrates coded as Al–HCl–H_2_O–BT–SA was prepared by sequential treatment with HCl, boiling water, bis-(γ-triethoxysilylpropyl)-tetrasulfide (KH-Si69, BT) and stearic acid (SA). The contrast samples coded as Al–HCl–BT–SA, Al–HCl–H_2_O–SA, and Al–HCl–SA were also prepared similarly by omitting the treatment in boiling-water, the BT passivation, and the treatment in boiling-water/passivation by BT, respectively. These samples were then immersed into an aqueous solution of NaCl with different flow velocity (0, 0.5, 1.0, 1.5, and 2.0 m s^−1^), and its dynamic corrosion behavior was investigated. The results showed that, as the flow velocity increased, the corrosion resistance of the Al–HCl–H_2_O–BT–SA sample indeed deteriorated. However, compared with the contrast samples of Al–HCl–BT–SA, Al–HCl–H_2_O–SA, and Al–HCl–SA, the deterioration in corrosion resistance for the Al–HCl–H_2_O–BT–SA sample was much lower, implying that the dynamic corrosion resistance of the superhydrophobic surfaces was closely related with the micro-structures and the organic passivated layers. The present study therefore provided a fundamental understanding for the applications of superhydrophobic samples to prevent the corrosion, especially, for various vessels in dynamic water.

## Abstract 2

For superhydrophobic surfaces immersed in water, a thin layer of air could be entrapped in the solid/liquid interface.

## Graphical abstract

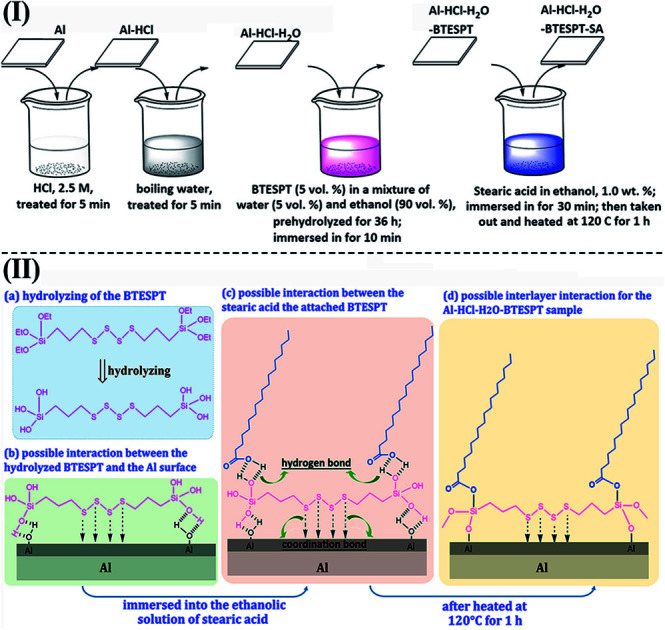

## 1. Introduction

Aluminum (Al), with many advantages such as low density, good ductility, high strength, good heat/electric conductivity, *etc.*, has been widely used in many fields such as electronics, aerospace, automobiles and so on. However, the application of Al is restricted because the Al surface often has localized corrosion in a medium of corrosive ions, such as Cl^−^, F^−^, Br^−^, I^−^, NO_3_^−^, SCN^−^, ClO_3_^−^, ClO_4_^−^, gluconate anions, and H^+^.^1–7^ Nowadays, chromate conversion coating with the characteristics of easy application and effectiveness is one of the most used adhesion promoters to improve the corrosion resistance for Al.^8^ However, the hexavalent chromium compounds used in chromate conversion coating is known to be highly carcinogenic and strongly toxic.^9,10^

Based on the need of environment protection, silane agents were applied as an alternative for chromates in the metal pretreatment industry.^11,12^ The silane agents with the hydrolysable alkoxy groups, such as Si–OMe and Si–OEt, undergo a hydrolysis process to produce silanols (Si–OH). Then, the Si–OH group can interact with the Al–OH group on the hydrated Al surface *via* the hydrogen bond. During the further thermal treatment, such bonds may convert to Al–O–Si and the silanes attach to the Al surface firmly. The excess Si–OH groups condense among themselves to form a siloxane network (Si–O–Si), which resists to aggressive species. However, the so-formed Al–O–Si and Si–O–Si bonds were susceptible to hydrolysis at high pH and the hydrolytic stability of these bonds was close to the molecular structures, such as the length of the alkyl chains^13^ and the type of terminal groups.^14–16^

**Fig. 1:**
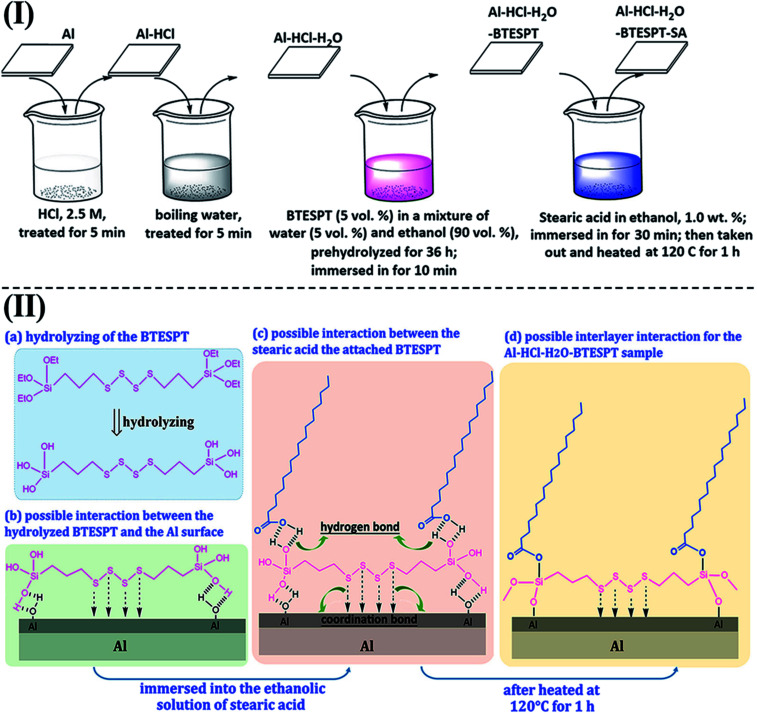
(I) The fabrication process of the Al–HCl–H_2_O–BT–SA sample; (II) possible mechanism for the formation of different samples.

Herein, a bis-silane, bis-(γ-triethoxysilylpropyl)-tetrasulfide (KH-Si69, BT), was used. Besides the hydrolysable alkoxy tail and head groups, the –S–S–S–S– group in the middle of the chain was able to enhance the hydrophobicity of the film, postponing water penetration during immersion process. To further improve the corrosion resistance of the BT films, many efforts have been made by adding dopants, such as rare earth cations^17–19^ and nanoparticles.^20–22^ Herein, we use another different way, *viz.*, constructing a hydrophobic outerlayer (stearic acid, SA) onto the BT film. To the best of our knowledge, it is not easy to graft the SA molecules to the hydrophobic BT film. However, the so-formed BT film without thermal treatment is hydrophilic with outer exposed Si–OH groups, which serve as the active sites to anchor the SA molecules [Fig. 1(II)].

On a rough-structured Al surface, we even obtained a BT–SA bilayer superhydrophobic film (abridged as SHF). As well known, the SHF is repellent to water with high water contact angle above 150° and sliding angle below 10°.^23–26^ Once the SHF is immersed into water, a layer of air will be entrapped in the solid/liquid interface, which can hinder the penetration of corrosive species (such as the Cl^−^ ions dissolved in water) to reach the metallic substrate and improve the corrosion resistance greatly.^27–31^ However, such studies^20–24^ were performed in the static aqueous solution. In the dynamic aqueous solution, the entrapped air on the SHF would be squeezed out much more easily and the longetivity of the entrapped air would be shortened to some extent. Correspondingly, the corrosion resistance due to the entrapped air would be weakened. Herein, to verify these assumptions, for the first time, the corrosion resistance of the so-obtained BT–SA SHF in the dynamic NaCl aqueous solution is investigated. It is expected that the present study can provide a fundamental understanding for the applications of superhydrophobic surfaces to prevent the corrosion, especially, for various vessels in dynamic water.

## 2. Experimental

### 2.1. Sample preparations

The Al was firstly ground with abrasive papers (600 # and 2000 #) and then ultrasonically cleaned in ethanol for 10 min. Then, the Al sample was treated sequentially by HCl, boiling water, bis-(γ-triethoxysilylpropyl)-tetrasulfide (KH-Si69, BT) and stearic acid (SA). The fabrication details and possible mechanisms were shown in Fig. 1. For convenience, the so-obtained sample was coded as Al–HCl–H_2_O–BT–SA. The fabrication procedure of the contrast superhydrophobic samples was the same but certain treatment was omitted. For instance, the treating in boiling-water and the BT passivation was omitted for the Al–HCl–BT–SA sample and the Al–HCl–H_2_O–BT–SA sample. For the Al–HCl–SA sample, both the treating in boiling-water and the passivation by BT was omitted.

### 2.2. Sample characterizations

An optical contact angle meter (Easydrop, Krüss, Germany) with a computer controlled liquid dispensing system and a motorized tilting stage was used to measure the contact angle at room temperature (25 °C) with ultrapure water (7 μL). The average contact angle values were obtained by measuring the sample at 5 different positions of the substrate. The morphological microstructures were observed on field emission scanning electron microscope (FE-SEM, JSM-6701F) under vacuum environment and the chemical compositions were characterized by energy dispersive X-ray spectroscopy (EDS, INCA 250, Oxford, UK). The surface chemical compositions was also measured by the X-ray photoelectron spectroscopy (XPS, Physical Electronics, PHI-5702, USA). Average thickness of the so-generated hydroxide in boiling water was measured by a DEKTAK-150 step profiler (Veeco Instruments Inc., United States) at 5 different positions. The measurements were performed using a monochromated Al-Kα irradiation and the chamber pressure was about 3 × 10^−8^ torr under testing condition. The binding energy of adventitious carbon (C1s: 284.8 eV) was used as a basic reference. The corrosion resistance in the NaCl aqueous solution (3.5 wt%) was evaluated by monitoring the change in surface wettability, surface morphology, and surface chemistry.

## 3. Results and discussion

### 3.1. Surface morphology, chemistry, and wettability

**Fig. 2:**
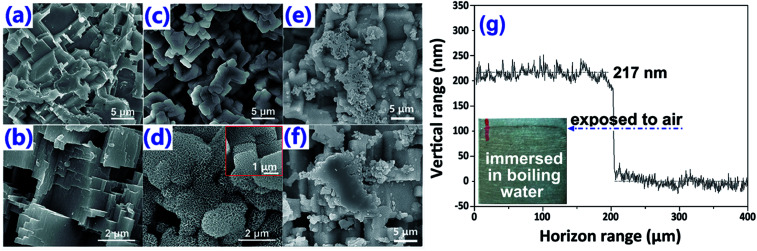
Surface morphology for the Al–HCl sample (a and b) and the Al–HCl–H_2_O sample (d–f). For (c) and (d), the treating time in the boiling water is 5 min; for (e) and (f), the treating time in the boiling water is 10 min and 15 min, respectively. One dimensional surface shape across the boundary of the boiling water treated and non-treated area (g); the measurements were performed at five different lines and the average thickness of the hydroxide was 205 ± 25 nm.

After chemically etched by HCl, micro-steps were formed on the surface [Fig. 2(a)]. As explained by Shen *et al.*, the formation of such micro-steps was due to the dislocation defects in the Al substrate with relatively higher energy, which were prone to be dissolved under the attack of chemical etchants, such as HCl.^32^ Due to the capillary effect, the so-formed Al–HCl sample was superhydrophilic (Table 1, sample a). After surface passivation by stearic acid (SA), the Al–HCl–SA sample turned to be superhydrophobic (Table 1, sample e). As discovered by Zhou *et al.*, the superhydrophobicity can further be enhanced by the nano-structures on the micro-steps.^33^ So, the Al–HCl sample was transferred into the boiling water to generate nano-flakes [Fig. 2(d)] and the so-formed sample was coded as Al–HCl–H_2_O. The optimal treating time in the boiling water to form the nano-flakes was determined to be 5 min. As the treating time prolonging to 10 min [Fig. 2(e)] or 15 min [Fig. 2(f)], some nano-flakes agglomerated and the sliding angle increased to 15.2 ± 1.2° or 25.1 ± 1.5° (Table 1, sample g). In the following text, if there is no other special instruction, the treating time in the boiling water for the Al–HCl–H_2_O sample and its derivatives was 5 min. As revealed in our previous study^34^ and other research,^35^ the so-formed nano-flakes are hydroxide of AlO(OH), which can serve as the barrier layer to improve the corrosion resistance. The thickness of the so-formed nano-flakes was determined to be 205 ± 25 nm by a step profiler.

**Table 1:** Surface wettability for different samples

Sample	Water contact angle (deg)	Sliding angle (deg)
a, Al–HCl	∼0	—
b, Al–HCl–H_2_O	∼0	—
c, Al–HCl–H_2_O–BT^a^	62.8 ± 1.5/155.7 ± 1.6	—/14.5 ± 0.9
d, Al–HCl–H_2_O–BT–SA^b^	167.9 ± 1.8/161.3 ± 1.6	3.6 ± 0.5/9.5 ± 1.2
e, Al–HCl–SA	166.3 ± 1.5	6.8 ± 0.8
f, Al–HCl–BT–SA	166.6 ± 1.9	7.2 ± 0.8
g, Al–HCl–H_2_O–SA^c^	166.2 ± 1.6/160.3 ± 2.1/156.7 ± 2.0	4.0 ± 0.4/15.2 ± 1.2/25.1 ± 1.5

a The former and latter value is for the unannealed and annealed sample, respectively.

b The former and latter value is for the sample prepared by immersing the unannealed and annealed Al–HCl–H_2_O–BT sample into the stearic acid, respectively, and then annealed at 120 °C.

c The first, second, and third value is for the Al–HCl–H_2_O sample with a treating time in the boiling water of 5 min, 10 min, and 15 min, respectively.

**Fig. 3:**
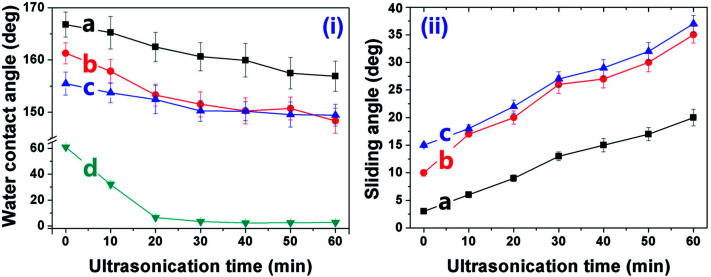
Variation of water contact angle and sliding angle against ultrasonication time in ethanol for the Al–HCl–H_2_O–BT–SA sample (a), the (Al–HCl–H_2_O–BT)_an_–SA sample (b), the Al–HCl–H_2_O–BT sample (c), and the unannealed Al–HCl–H_2_O–BT sample (d).

The AlO(OH) species are abundant with hydroxyl groups, which may facilitate the anchoring of silanes, such as the bis-(γ-triethoxysilylpropyl)-tetrasulfide (BT). As revealed in other research,^35^ the so-hydrolyzed BT is attached to the Al (110) surface *via* the chemical adsorption of the sulfur atoms with the aluminum ones and the hydrogen bonding between the so-formed Si–OH and the Al–OH bonding [Fig. 1(II)(b)]. However, it is supposed that only one out of three Si–OH bonding is interacted with the substrate; the other two are exposed outside. This suggests that there are some Si–OH groups on the surface; consequently, the Al–HCl–H_2_O–BT sample is hydrophilic with the contact angle of 62.8 ± 1.5° (Table 1, sample c). Under further annealing, the outer exposed Si–OH groups may turn into the Si–O–Si ones and the surface was dominated by the groups with low-surface-energy, such as –CH_2_– and –S–S–S–S–. Thus, the contact angle and sliding angle of the annealed Al–HCl–H_2_O–BT sample turned to be approx. 155.7° and approx. 14.5°, respectively (Table 1, sample c). Moreover, under annealing, the hydrogen bonding between the Al–OH group and the Si–OH group may also converted into the Al–O–Si chemical bonding,^36,37^ and the BT molecules are attached to the substrate more firmly. To prove this assumption, the annealed and the unannealed Al–HCl–H_2_O–BT samples were immersed into the ethanol under ultrasonication and the variation of surface wettability was monitored (Fig. 3). After 20 min, the contact angle for the unannealed Al–HCl–H_2_O–BT sample (curve d in Fig. 3) decreased sharply to approx. 5° and the annealed sample (curve c in Fig. 3) still possessed a contact angle above 150°.

We also performed a contrast experiment to infer that the exposed Si–OH groups on the un-annealed Al–HCl–H_2_O–BT sample were essential for the anchoring of SA. The (Al–HCl–H_2_O–BT)_an_–SA sample was prepared by immersing the annealed Al–HCl–H_2_O–BT sample without Si–OH groups into the ethanol solution of SA and then annealed at 120 °C. The so-obtained (Al–HCl–H_2_O–BT)_an_–SA sample possessed a high contact angle of 161.3 ± 1.6° and low sliding angle of 9.5 ± 1.2° (the latter data for sample d in Table 1), which were superior to the data (155.7 ± 1.6°and 14.5 ± 0.9°, the latter data for sample c in Table 1) for the annealed Al–HCl–H_2_O–BT sample, however, still inferior to the data (167.9 ± 1.8° and 3.5 ± 0.5°, the former data for sample d in Table 1) for the Al–HCl–H_2_O–BT–SA sample. Although the superhydrophobicity was achieved for the (Al–HCl–H_2_O–BT)_an_–SA sample, the anchoring of SA molecules to the (Al–HCl–H_2_O–BT)_an_ surface was supposed to be weak and would be detached easily. To confirm this, the (Al–HCl–H_2_O–BT)_an_–SA sample (curve b in Fig. 3) was ultrasonicated in ethanol. After merely 20 min ultrasonication in ethanol, the curves for samples of the (Al–HCl–H_2_O–BT)_an_ and (Al–HCl–H_2_O–BT)_an_–SA almost overlapped. This happened because the SA molecules were removed from the (Al–HCl–H_2_O–BT)_an_–SA sample and its wettability was similar to that of the (Al–HCl–H_2_O–BT)_an_ sample. However, for the Al–HCl–H_2_O–BT–SA sample, the interaction between the BT layer and the SA molecules was supposed to be the covalent C–O–Si bonding (Fig. 1(II)d), so the Al–HCl–H_2_O–BT–SA sample (curve a in Fig. 3) possessed much better excellent durability against the ultrasonication as compared with the (Al–HCl–H_2_O–BT)_an_–SA sample (curve b in Fig. 3).

**Fig. 4:**
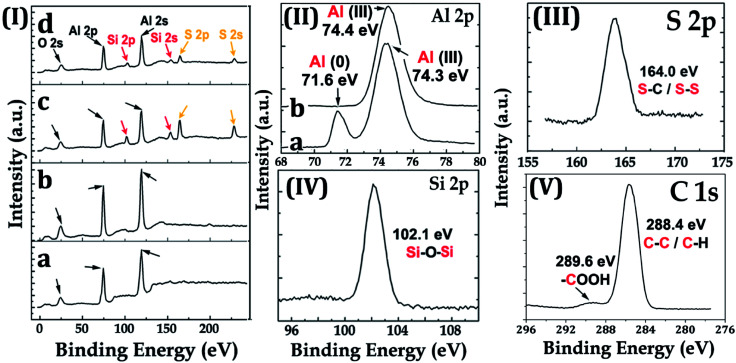
X-ray photoelectron spectra for the Al–HCl sample (I)-(a), (II)-(a), the Al–HCl–H_2_O sample (I)-(b), (II)-(b), the Al–HCl–H_2_O–BT (I)-(c), (III), (IV), and the Al–HCl–H_2_O–BT–SA sample (I)-(d), (V).

The surface chemistry of the samples was evaluated by XPS. For the Al–HCl sample, the Al (iii) signal in the Al 2p spectrum [Fig. 4(II)(a)] was unexpectedly visible. It was supposed that, in the aqueous solution of HCl, the oxide layer would be eliminated through the chemical reaction as shown in eqn (1). Thus, there should have been no oxide species and the related Al (iii) signal. However, once the Al–HCl sample was taken out and exposed to the air, the outermost Al (0) species would be oxidized into Al_2_O_3_ easily (eqn (2)). Consequently, the Al (iii) signal emerged. The visible Al (0) signal in Fig. 4(II)(a) also suggested that the newly-formed oxide layer was thin and the Al (0) beneath it was still detectable. For the Al–HCl–H_2_O sample, as revealed in other research,^21–23^ the boehmite of AlO(OH) was formed through the chemical reactions as shown in eqn (3).

1 Elimination of oxide by HCl : Al_2_O_3_ + 6HCl → 2AlCl_3_ + 3H_2_O

2 Formation of oxide in the air : 4Al(0) + 6O_2_ → 2Al_2_O_3_

3
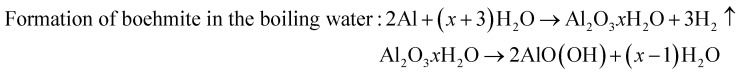


For the Al–HCl–H_2_O–BT sample, Si 2s/2p and S 2s/2p peaks attributed to the Si and S atoms, respectively, in the BT molecules were observed. For the Al–HCl–H_2_O–BT–SA sample, the C 1s spectrum showed a small peak at 289.6 eV, which was attributed to the –COOH group in the SA molecule. Moreover, due to the overlayed SA molecules, the peaks originated from BT sublayer became weaker, however, still visible [comparing the related peaks in Fig. 4(I)(c) and (I)(d)].

### 3.2. Corrosion resistance in the NaCl solution

**Fig. 5:**
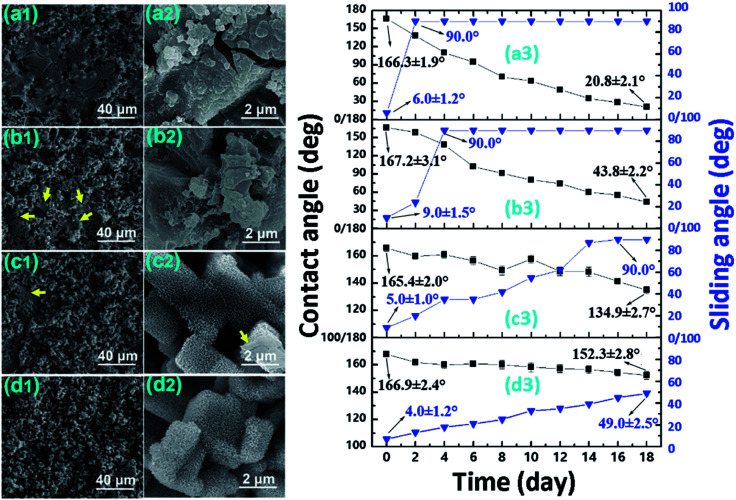
Surface morphology for different sample ((a), the Al–HCl–SA sample; (b), the Al–HCl–BT–SA sample; (c), the Al–HCl–H_2_O–SA sample; (d), the Al–HCl–H_2_O–BT–SA sample) in the static NaCl aqueous solution for 18 days and variation of surface wettability against immersion time.

To evaluate the corrosion resistance, the variation in surface wettability (Fig. 5), surface morphology (Fig. 5), and surface chemistry (Table 2) was monitored. As shown in Fig. 5(a3), after only 2 days in the static NaCl aqueous solution, the sliding angle for the Al–HCl–SA sample increased to 90°. In this condition, the water droplet adhered to the surface firmly even when the sample was laid vertically or upside-down. Once the BT interlayer was added, the time taken for the sliding angle increasing to 90° prolonged to 4 days [(Fig. 5(b3))]. For the Al–HCl–H_2_O–SA sample, the corresponding time prolonged largely to 16 days [(Fig. 5(c3))]. For the Al–HCl–H_2_O–BT–SA sample, even after 18 days, the contact angle was still as high as 152.3 ± 2.8° and the sliding angle was 49.0 ± 2.5° [(Fig. 5(d3))]. The surface morphology for different samples after static corrosion for 18 days was also examined by FE-SEM. For the Al–HCl–SA sample [Fig. 5(a1) and (a2)], the nano-flakes became compacted and lots of micro-sheets were formed atop. For the Al–HCl–BT–SA sample [Fig. 5(b1) and (b2)], fewer micro-sheets were observed as compared with the Al–HCl–SA sample. For the Al–HCl–H_2_O–SA sample, only some compact micro-structures instead of the micro-sheets were observed (as indicated by the yellow arrows in Fig. 5(c1). Under higher magnification, it was clearly observed that the nano-flakes on a micro-step became compacted (as indicated by the yellow arrows in Fig. 5(c2)). For the Al–HCl–H_2_O–BT–SA sample, the change in surface morphology was so slight and we could not tell the difference between Fig. 5(d2) and 2(d). So, we could say that the Al–HCl–H_2_O–BT–SA sample possessed the best corrosion resistance in the static NaCl aqueous solution.

**Table 2:** Content ratio of oxygen to aluminum before and after corrosion (*viz.*, *R*_[O]/[Al],bc_ and *R*_[O]/[Al],ac_) and the increasing ratio (IR) calculated by the eqn (7)

Sample	*R*_[O]/[Al],bc_	Static corrosion, 18 d		Dynamic corrosion, 2.0 m s^−1^, 50 h	
		*R*_[O]/[Al],ac_	IR	*R*_[O]/[Al],ac_	IR
Al–HCl–SA	0.05	1.49	2880.0%	0.49	880.0%
Al–HCl–BT–SA	0.07	0.94	1242.9%	0.42	500.0%
Al–HCl–H_2_O–SA	0.73	1.15	57.5%	1.01	38.4%
Al–HCl–H_2_O–BT–SA	0.84	0.98	16.7%	0.91	8.3%

**Fig. 6:**
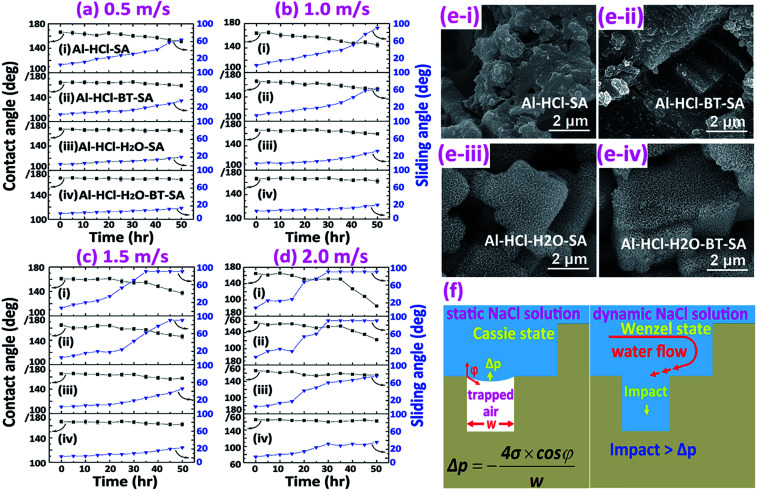
Variation of surface wettability against immersion time in NaCl aqueous solution with different flow velocity of 0.5 m s^−1^ (a), 1.0 m s^−1^ (b), 1.5 m s^−1^ (c), and 2.0 m s^−1^ (d). Surface morphology for different sample immersed in NaCl aqueous solution with the flow velocity of 2.0 m s^−1^ for 50 h (e). Mechanism for the corrosion resistance of the superhydrophobic surface in the static and the dynamic NaCl aqueous solution (f).

Similarly, the corrosion resistance in the dynamic NaCl aqueous solution was also estimated by monitoring the variation in surface wettability (Fig. 6a–d) and surface morphology (Fig. 6e). It was obvious that, as the velocity of water flow increased from 0.5 m s^−1^ to 2.0 m s^−1^, the deterioration of superhydrophobicity became faster (Fig. 6a–d). In other words, as the flow velocity increased, the water contact angle decreased and the sliding angle increased more quickly. This phenomenon could be well understood if one noticed that, in the dynamic NaCl aqueous solution, an impact proportional to the flow velocity as shown in Fig. 6f would be formed on the uneven solid surface. The so-formed impact would accelerate the expellation of trapped air from the water/solid interface and the transition from Cassie wetting state to Wenzel state. In the Wenzel wetting state, the NaCl aqueous solution was in contact with the aluminum directly; consequently, the corrosion was more seriously and the deterioration of superhydrophobicity became faster. We also observed that the deterioration of superhydrophobicity for the Al–HCl–H_2_O derived superhydrophobic samples was much slower as compared with the Al–HCl derived superhydrophobic samples. This was mainly because that, for the Al–HCl–H_2_O sample, the micro-steps were covered by nano-flakes. As shown in the equation in Fig. 6f, the resistance to the impact was in inverse proportional to the scale of micro-/nano- structures. In other words, even if the trapped air among the micro-steps was expelled, the trapped air among the nano-flakes might remain stable to slow down the corrosion and the deterioration of superhydrophobicity. For the Al–HCl–H_2_O–BT–SA sample, it possessed the best corrosion resistance. Even after immersed in the 2 m s^−1^ NaCl aqueous solution for 50 h, the contact angle for the Al–HCl–H_2_O–BT–SA sample still remained above 150° (Fig. 6d) and the surface micro-structures were not destroyed obviously (Fig. 6e(iv)).

4 Hydration of boehmite : AlO(OH) + H_2_O → Al(OH)_3_

5
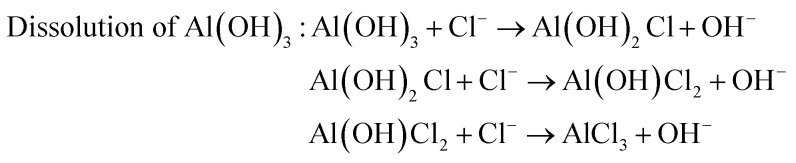
6
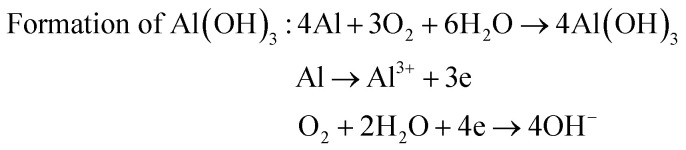


The surface chemistry was measured by EDX and the content ratio of oxygen to aluminum, *viz.*, [O]/[Al], was used to estimate the corrosion.^38^ The corrosion process included a series of chemical and electrochemical reactions. Once the water penetrated through the organic layer, the boehmite would be hydrated (eqn (4)) to Al(OH)_3_, which would further be dissolved gradually under the attack of Cl^−^ anion (eqn (5)). Once the boehmite was dissolved completely, aluminum matrix would be exposed directly to the NaCl solution and would further be converted to Al(OH)_3_ through electrochemical corrosion reactions (eqn (6)). Due to the relatively higher oxygen content in the corrosive product of Al(OH)_3_ as compared with AlO(OH), after corrosion, the content ratio of oxygen to aluminum, *viz.*, [O]/[Al], increased (Table 2). The increasing ratio (IR) was simply calculated by the eqn (7)7
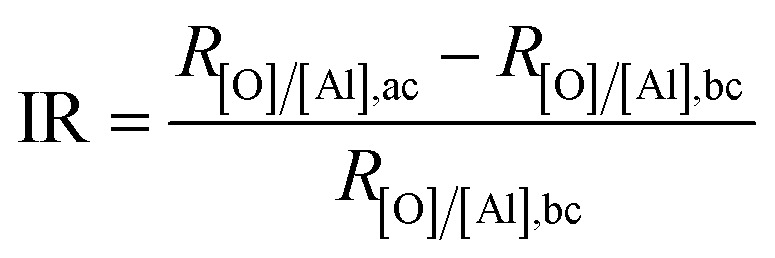
where *R*_[O]/[Al],bc_ and *R*_[O]/[Al],ac_ was the [O]/[Al] value before and after corrosion, respectively.

For the Al–HCl derived samples, the oxide layer was removed by HCl and the oxygen content as expressed by *R*_[O]/[Al],bc_ was much smaller as compared with the Al–HCl–H_2_O derived samples. Meanwhile, due to the absence of the barrier oxide layer, after static corrosion for 18 days, the *R*_[O]/[Al],ac_ for the Al–HCl–SA sample and the Al–HCl–BT–SA sample increased greatly (Table 2). Specifically, for the Al–HCl–SA sample, the IR value was as high as 2880.0%, which decreased to 1242.9% once the BT interlayer was added. We also noticed that, the IR values for the Al–HCl–H_2_O derived superhydrophobic samples were much smaller than the corresponding Al–HCl derived superhydrophobic samples. This suggested that the BT molecules and the boehmite layer could enhance the corrosion resistance of the superhydrophobic sample greatly. Similarly, we found that once the BT molecules or/and oxide layer was added, the IR values in Table 2 decreased greatly. In other words, the BT molecules and oxide layer could retard the corrosion process and consequently the variation in surface chemistry was small. All these findings proved that the oxide layer and BT layer could enhance the corrosion protection in both static and dynamic NaCl aqueous solution greatly.

## 4. Conclusions

For the first time, the dynamic corrosion of superhydrophobic surface was investigated by monitoring the variation in surface wettability, surface morphology, and surface chemistry. Results showed that, as the flow velocity of NaCl aqueous solution increased, the corrosion resistance of the Al–HCl–H_2_O–BT–SA sample deteriorated. This was probably because the retained air layer in the solid/liquid interface was less stable in the dynamic NaCl aqueous solution. However, compared with the contrast samples, such as the Al–HCl–BT–SA sample, the Al–HCl–H_2_O–SA sample, and the Al–HCl–SA sample, the deterioration in dynamic corrosion resistance for the Al–HCl–H_2_O–BT–SA sample was much lower. This suggested that the dynamic corrosion of superhydrophobic surface was closely related with the micro-structures and the organic passivated layer. To sum up, as the water flow velocity increased, the corrosion resistance of the superhydrophobic surface indeed deteriorated; however, by rational surface designing (such as the surface structures with smaller dimension and the passivated layer with better corrosion resistance), such deterioration could be slowed down. The present study therefore provided a fundamental understanding for the applications of superhydrophobic samples to prevent the corrosion, especially, for various vessels in dynamic water.

## Conflicts of interest

There are no conflicts to declare.

## Supplementary Material
